# A method to design polarization reconfigurable antenna with simple switching mechanism and compact size characteristics

**DOI:** 10.1038/s41598-025-97908-1

**Published:** 2025-04-18

**Authors:** Thai Nguyen-Dinh, Tan Dao-Duc, Dinh Nguyen-Quoc, Hung Tran-Huy, Niamat Hussain

**Affiliations:** 1 Faculty of Radio Electronics Engineering, Le Quy Don Technical University, Hanoi, 11917 Vietnam; 2 Faculty of Electrical and Electronic Engineering, PHENIKAA University, Hanoi, 12116 Vietnam; 3 Vietnam-Japan International Cooperation Center for Science and Technology (VJIC), Le Quy Don Technical University, Hanoi, 11917 Vietnam; 4 Electronics and Nanoscale Engineering, University of Glasgow, Glasgow, UK

**Keywords:** Electrical and electronic engineering, Engineering

## Abstract

A reconfigurable antenna with simple polarization switching mechanism and compact size characteristics is presented in this paper. The primary radiating aperture of the proposed design is four parasitic elements arranged in a 2 $\times$ 2 configuration. This radiating aperture is coupled with an excitation source whose polarizations can be controlled by a switchable feeding network. By employing only two PIN diodes, the proposed antenna can produce unidirectional beams with different polarizations, including linearly polarized (LP), left-hand and right-hand circularly polarized (LHCP, RHCP) modes. It is worth mentioning that employing a switchable feeding network helps to minimize the negative effect of the biasing circuit to the antenna operation characteristics. Experimental results indicate that the consistent operational bandwidth across all states spans from 2.44 to 2.47 GHz. Furthermore, the recorded broadside gain reaches approximately 4.8 dBi. In comparison with existing studies, the proposed antenna achieves multiple polarization configurations while utilizing the least number of diodes and maintaining a compact design.

## Introduction

In contemporary wireless communication, antennas with polarization reconfigurability have garnered significant interest due to their capability to improve signal quality and system performance^1,2^. Microstrip patch antennas are extensively utilized in applications that demand high gain and a directional radiation pattern. Various patch antenna designs incorporating different polarization switching techniques have been introduced in^3–9^. However, these antenna structures are capable of switching their polarizations between left-hand and right-hand circularly polarized (LHCP and RHCP) states. For both CP and LP realizations, the design in^10–14^ proposed different approaches to achieve dual-sense CP, single or dual LP radiations. Here, the diodes and biasing circuit are directly attached to the polarization reconfigurable radiating element. As the diodes work as ON/OFF switches, the current distribution on the patch can be altered, enabling different polarization states. Nevertheless, a major limitation of integrating PIN diodes directly into the radiating structure is the unintended radiation from the diodes and the biasing circuit, which can adversely impact the antenna’s radiation characteristics.

To address this limitation, an alternative approach for polarization reconfigurability is to utilize switchable feeding networks^15–19^. Dual-sense CP modes are achieved in^15^ by utilizing several diodes and passive components, such as resistors, inductors and capacitors. Meanwhile, a similar performance is attained in^16^ and^17^ while utilizing fewer diodes. Additionally, an enhanced capability in terms of the number of polarization states is observed in^18,19^. However, these designs are constrained by the drawback of necessitating a substantial number of PIN diodes and relatively large antenna dimensions.

Although many polarization reconfigurable antennas have been reported, their limitations are the complicated switching mechanism and bulky size structure. This paper presents a polarization reconfigurable antenna with a small number of diodes and compact size as well. The compact feature is achieved by using four square-rings as the radiating aperture. Meanwhile, polarization reconfigurability is achieved by coupling the radiating aperture with the excitation source with its polarization is controlled by two PIN diodes on the T-junction divider.The antenna is first characterized using simulation tool and then confirm with measurement.

## Passive antenna

**Fig. 1:**
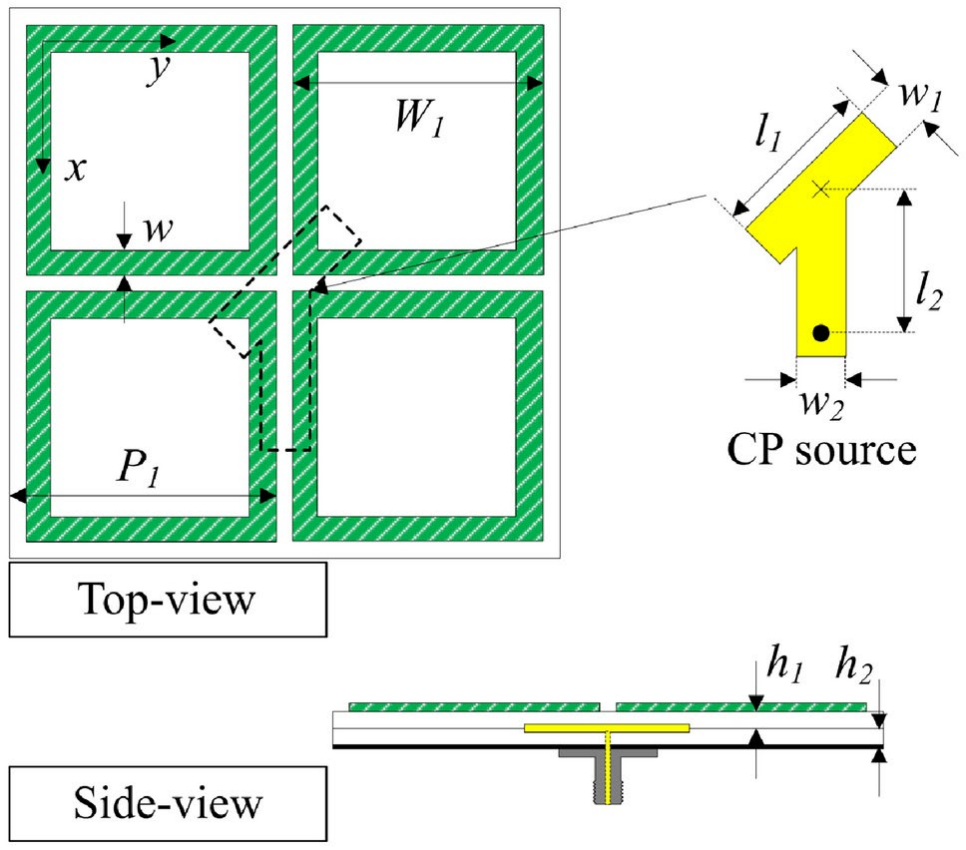
Geometrical configuration of the proposed passive antenna. The design parameters are $P_{1} = 22$, $W_{1} = 20$, $w = 1.7$, $l_{1} = 13.2$, $w_{1} = 3.8$, $l_{2} = 12.4$, $w_{2} = 4$, $h_{1} = h_{2} = 1.52$ (unit: mm).

This paper focuses on designing the compact polarization reconfigurable antenna. Thus, the primary radiating aperture should be thoroughly considered. Basically, the performance of a conventional patch antenna strongly depends on the ground plane size. For an antenna with a small ground plane, the high diffraction of the surface wave at the edges of the ground significantly affects the forward and backward radiations. Here, low forward radiation and high backward radiation are obtained. Thus, when the size is fixed, the method to improve the antenna gain is to increase the radiating aperture. Figure 1 shows the geometrical configuration of the compact CP antenna. Here, four square rings are employed as a primary radiating aperture of the antenna. This aperture is excited through a Y-shaped patch, which can produce two orthogonal fields with equal magnitude and 90 degree phase difference. It is noteworthy that, in comparison with the square-shaped radiating aperture presented in^20^, the adoption of a ring-shaped design contributes to an approximate 36% reduction in overall dimensions. The antenna is constructed using two Taconic RF-35 substrates, each with a thickness of 1.52 mm.

**Fig. 2:**
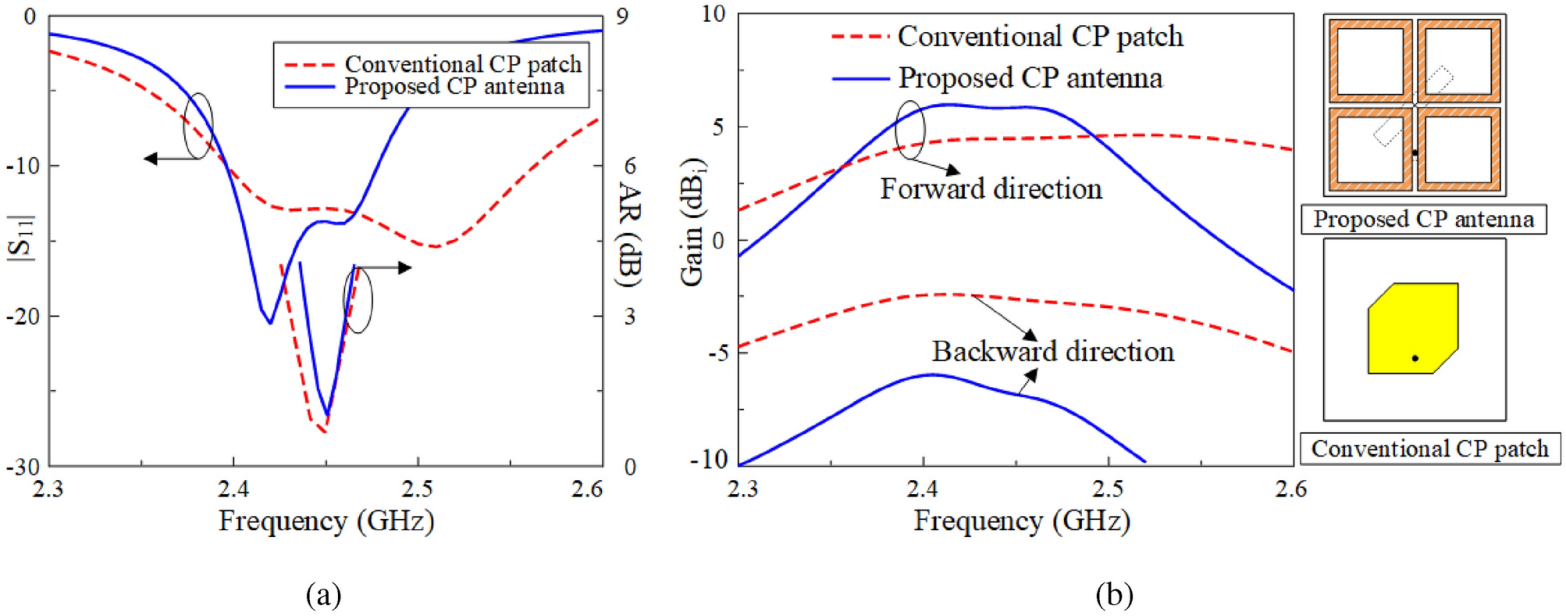
Simulated results of (**a**) $|S_{11}|$, AR and (**b**) gain of the proposed passive antenna.

**Fig. 3:**
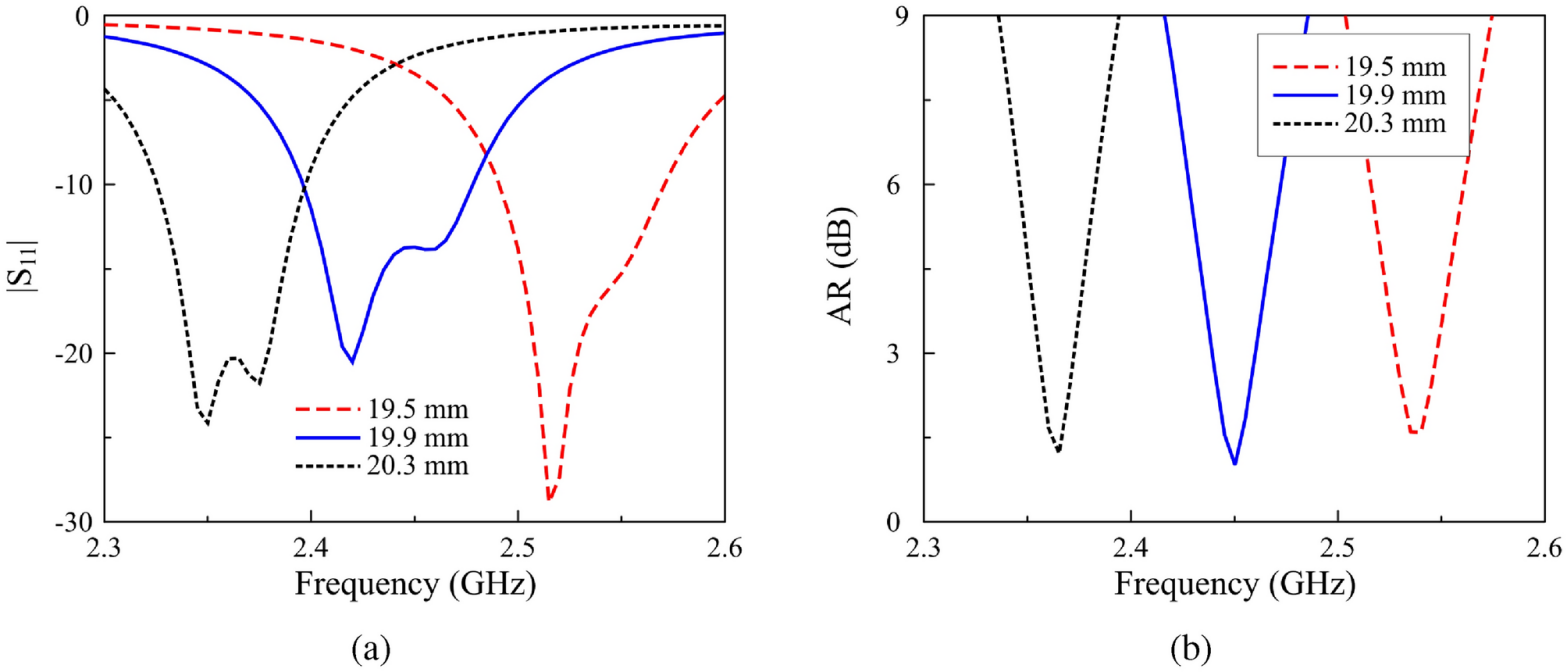
Simulated results of (**a**) $|S_{11}|$ and (**b**) AR for the antenna with varying $W_1$ values.

The simulated results for the proposed passive antenna, including the reflection coefficient $|S_{11}|$, axial ratio (AR), and broadside realized gain, are illustrated in Fig. 2. The data is also compared with a conventional truncated-corner CP patch. As seen, both antennas work at 2.45 GHz with similar matching and AR performance. However, the primary distinction lies in the gain levels in the backward and forward directions, as illustrated in Fig. 2b. Due to its larger radiating aperture, the proposed antenna exhibits stronger forward radiation. Additionally, the power is more efficiently coupled into the radiating aperture rather than being diffracted at the substrate edges, thereby significantly reducing back radiation. This characteristic highlights the advantage of the proposed design. Furthermore, it is noted that the operating frequency of the antenna is highly influenced by the dimensions of the ring-shaped patch ($W_1$), as demonstrated in Fig. 3. An increase in $W_1$ results in a downward shift of the operating band. Meanwhile, the AR and impedance matching can be conveniently adjusted by modifying the Y-shaped patch and the feed position.

## Antenna with polarization reconfigurable capability

### Antenna design

**Fig. 4:**
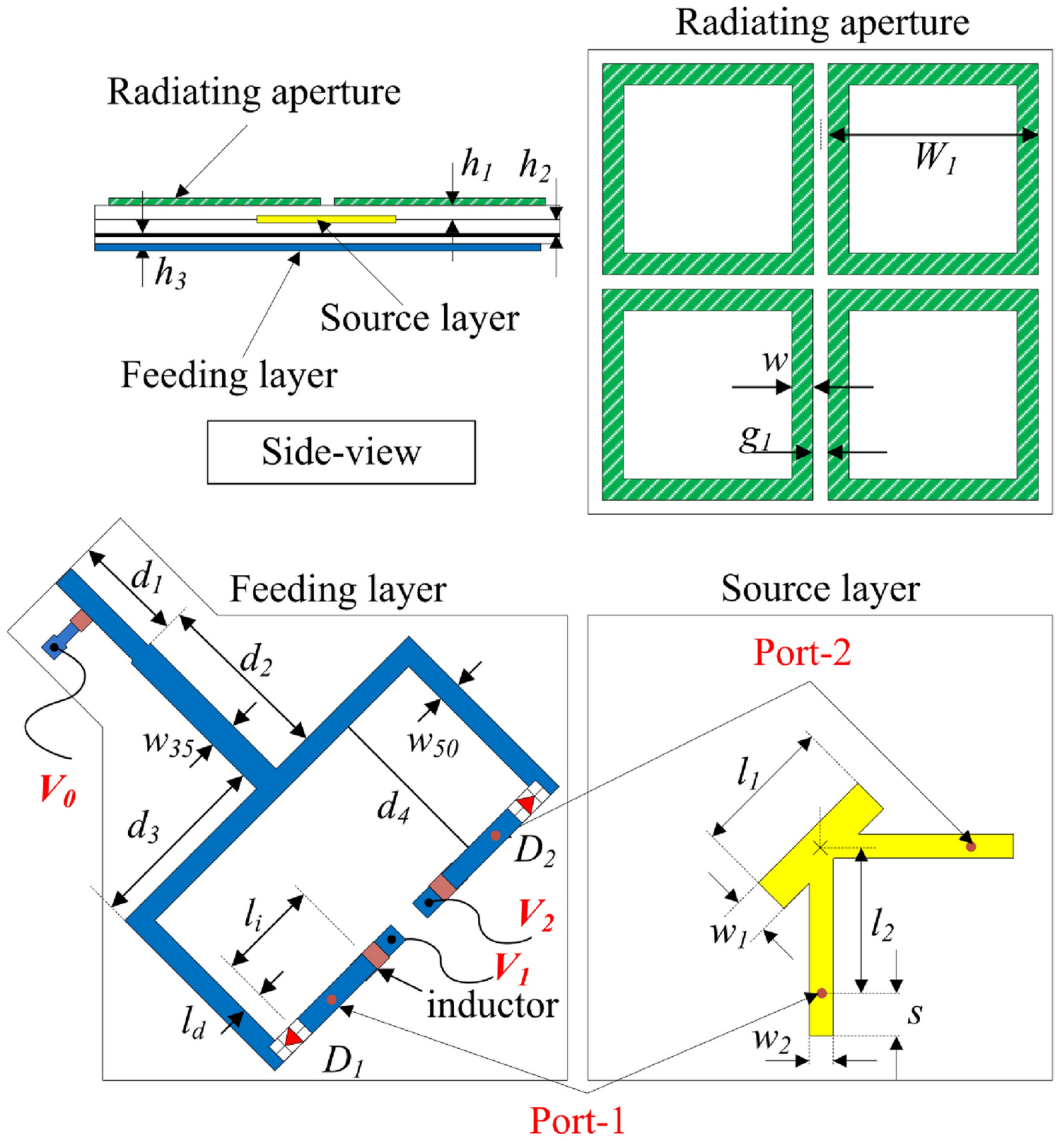
Structure of the proposed polarization-reconfigurable CP antenna.

In this section, a polarization reconfigurable antenna is introduced as an extension of the passive design. Figure 4 presents the geometry of different layers of the proposed reconfigurable structure. For dual-CP realization, the CP source has dual feeding positions, Port-1 and -2. When Port-1 is excited, the antenna emits an LHCP wave, whereas RHCP radiation is realized when Port-2 is excited. Furthermore, simultaneous excitation of both ports results in LP radiation. The excitation mechanism is implemented via a T-junction power fabricated on a FR4 substrate. The antenna dimensions are $g_{1}$ = 1.7, $W_{1}$ = 20.3, *w* = 1.9, $l_{1}$ = 12, $w_{1}$ = 3, $l_{2}$ = 12, $w_{2}$ = 1.2, *s* = 6, $w_{35}$ = 2.4, $w_{50}$ = 1.9, $d_{1}$ = 9.7, $d_{2}$ = 15.9, $d_{3}$ = 16.8, $d_{4}$ = 18.9, $l_{i}$ = 9.8, $l_{d}$ = 1.0, $h_{1}$ = $h_{2}$ = 1.52, $h_{3}$ = 0.8 (unit: mm).

To enable polarization diversity, two diodes, $D_{1}$ and $D_{2}$, are employed. These diodes belong to the MADP-042305-130600 series. In simulation, the ON and OFF states are respectively equivalent to 1.32 Ohm resistor and 0.15 pF capacitor^21^. The diodes are integrated into the T-junction power divider, and their specific placements are illustrated in Fig. 4. To achieve independent control of the diodes, distinct voltage levels, namely $V_{0}$, $V_{1}$, and $V_{2}$, are applied. Three inductors with value of 220 nH are used to isolate the divider to the biasing wires.

### Antenna operation

**Fig. 5:**
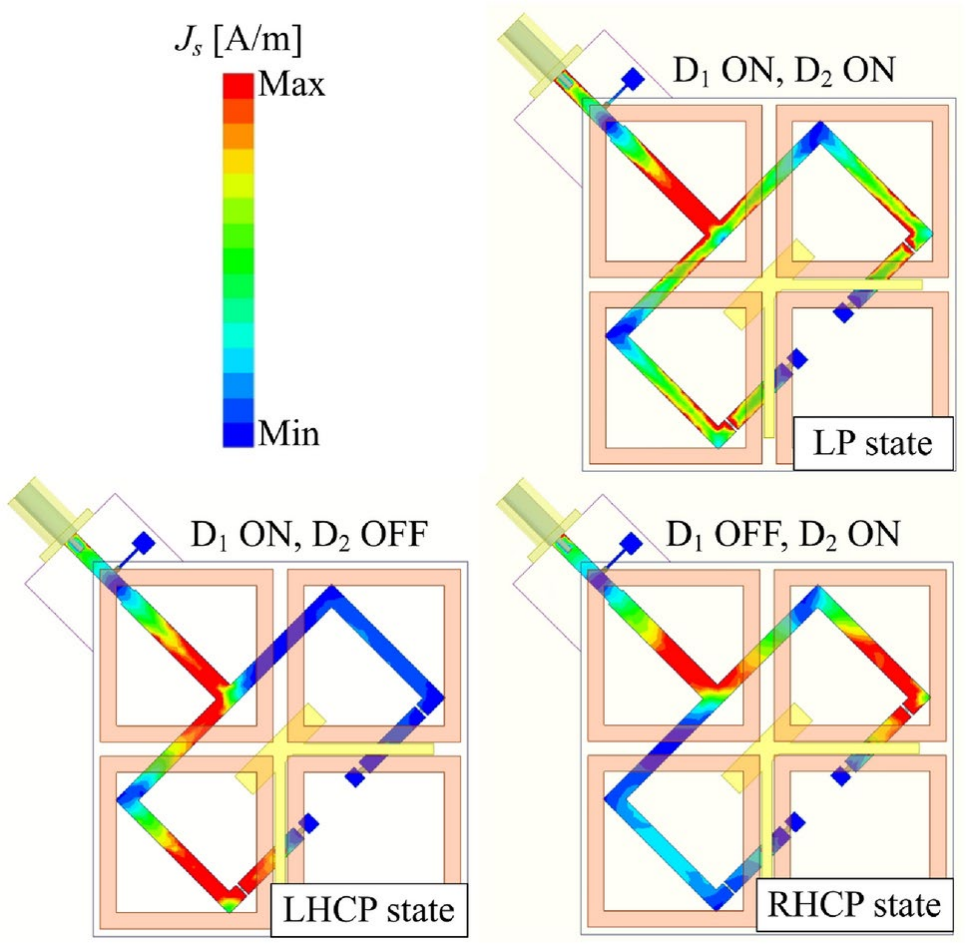
Simulated current distribution on the feeding network at 2.45 GHz.

Based on the diode operation described above, it is evident that when $D_{1}$ is in the ON state and $D_{2}$ is OFF, the primary source is fed through Port-1, resulting in LHCP radiation. Conversely, when $D_{2}$ is switched ON and $D_{1}$ is turned OFF, the primary source is fed through Port-2, generating RHCP radiation. Additionally, activating both $D_{1}$ and $D_{2}$ simultaneously enables excitations from both Port-1 and Port-2, producing LP radiation. A summary of the polarization states corresponding to different ON/OFF configurations of the PIN diodes is provided in Table 1. Besides, the current flowing on the divider is also investigated, as illustrated in Fig. 5. As observed, the RF current predominantly flows along the branch where the diode is in the ON state. Meanwhile, when both diodes are activated, the current is distributed across both branches.

**Table 1:** Operating states of the proposed antenna.

**Polarization**	**Controlled voltage**	$\hbox {D}_{1}$	$\hbox {D}_{2}$
LHCP	$V_{1} > V_{0}$	ON	OFF
RHCP	$V_{2} > V_{0}$	OFF	ON
LP	$V_{1} = V_{2} > V_{0}$	ON	ON

For antenna optimization, the AR performance is fine-tuned by adjusting the Y-shaped patch. Meanwhile, the impedance matching characteristics are largely influenced by the divider and the biasing circuit. A key factor in this optimization process is the placement of the diodes. As illustrated in Fig. 5, for CP realization, the current flowing into the output branch connected to $D_{1}$ is negligible when $D_{2}$ is in the ON state, and vice versa. On the other hand, the current flows equally to the output ports for the LP state. As a result, the positioning of the diodes plays a crucial role in determining the matching performance of the CP states. The effect of the output branch connected to the OFF-state diode on the other branch connected to the ON-state diode has to be minimized. The distance from the diode position to the T-divider output is expressed as $L = l_{d} + d_{3} + d_{4}$. For CP operation, assuming $D_{1}$ is ON while $D_{2}$ is OFF, the output from the T-divider connected to $D_{2}$ behaves as an open-ended stub, with an input impedance given by $Z_{in} = -jZ_{o} \cot (\beta L)$, where $\beta$ represents the phase constant and is defined as $\beta = {2\pi }/\lambda _{g}$, where $\lambda _{g}$ denotes the effective wavelength. To minimize the influence of this open-ended stub on the active port, $Z_{in}$ is set to infinity, which requires *L* to be approximately $\lambda _{g}/2$^22^.

**Fig. 6:**
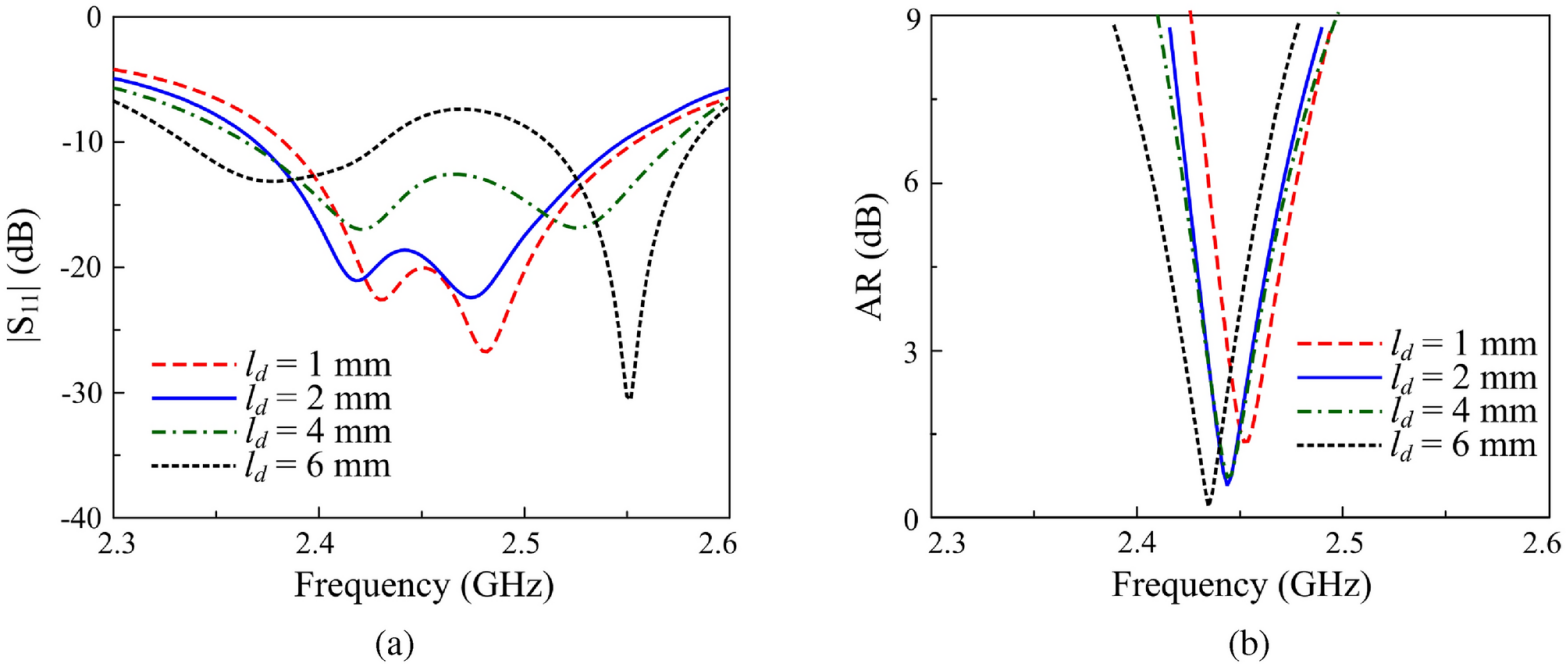
Simulated results of (**a**) $|S_{11}|$ and (**b**) AR for the proposed antenna in the LHCP state with varying diode position $l_d$.

**Fig. 7:**
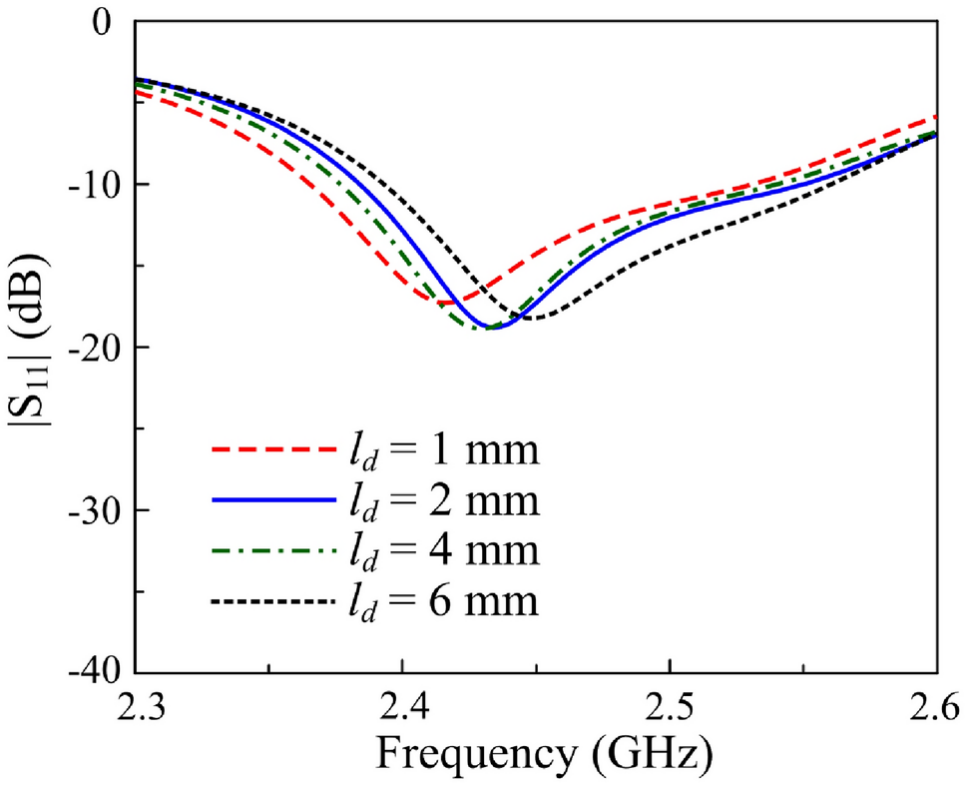
Simulated $|S_{11}|$ of the proposed antenna in the LP state with varying diode position $l_d$.

**Fig. 8:**
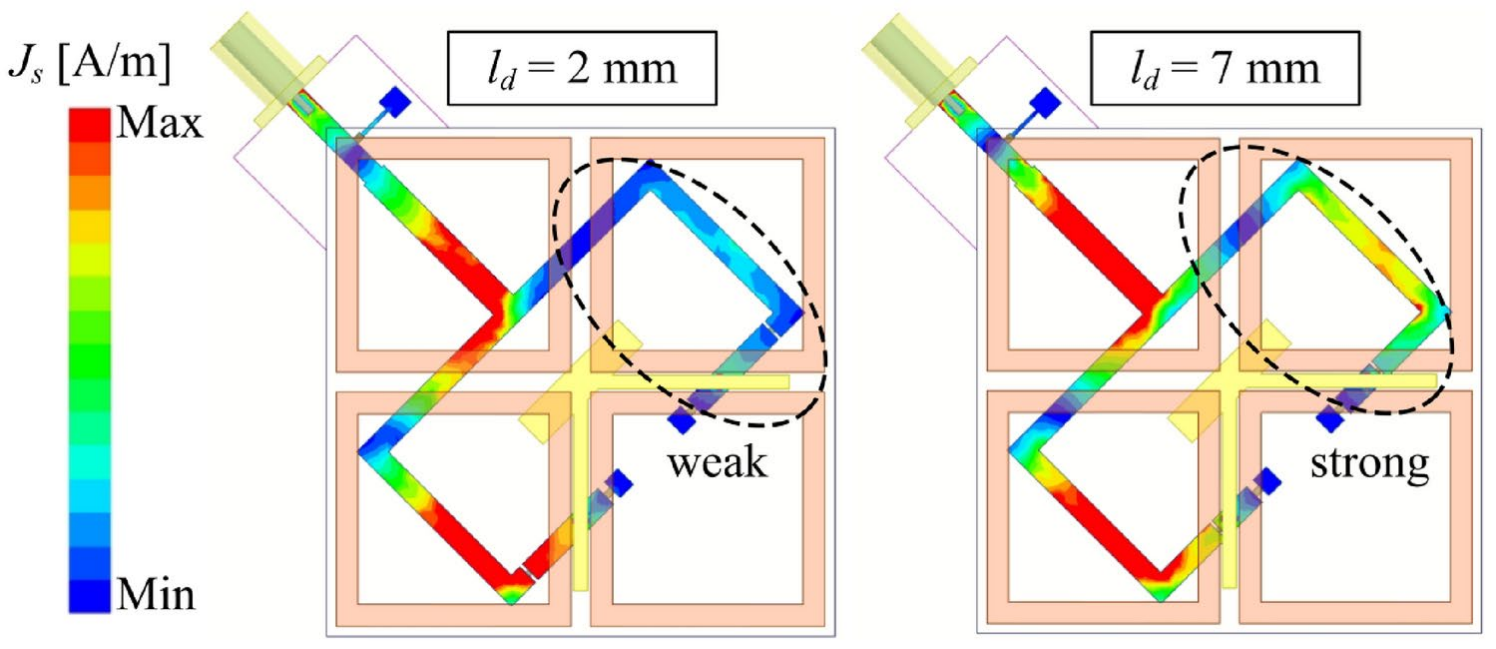
Simulated current distribution at 2.45 GHz for LHCP state.

To further validate this, the antenna performance for various diode positions ($l_{d}$) is analyzed in Figs. 6 and 7. The results indicate that the matching performance of the CP state is highly sensitive to changes in $l_{d}$. The optimal value of $l_{d}$ is determined to be 2 mm, corresponding to an *L* value close to half of the effective wavelength at 2.45 GHz. Conversely, variations in $l_{d}$ have a negligible impact on the LP state. The current distribution at 2.45 GHz for the LHCP state, as depicted in Fig. 8, further supports this observation. Specifically, when $l_{d} = 2$ mm, the current on the opposite branch remains weak, whereas increasing $l_{d}$ to 7 mm results in a significantly stronger current distribution. This is consistent with the theoretical explanation presented above.

**Fig. 9:**
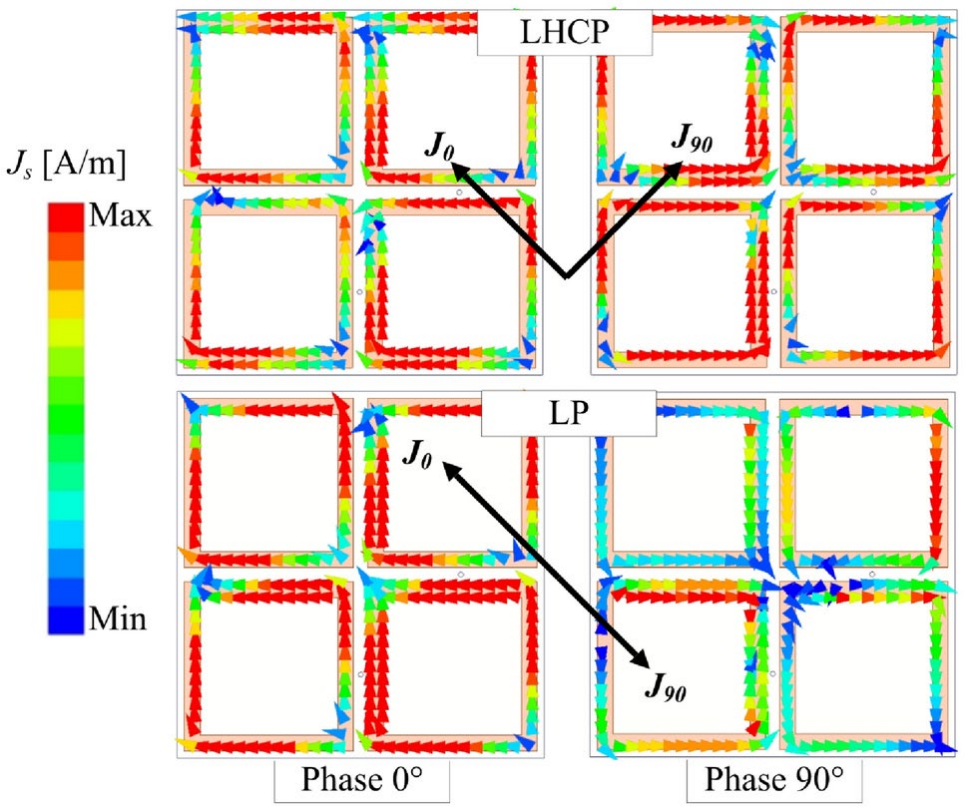
Simulated current distribution on the radiating aperture at 2.45 GHz.

Finally, the current distributions are used to verify the polarization realization. Figure 9 illustrates the surface current behavior on the radiating aperture at 2.45 GHz. The data confirms the LHCP and LP realizations. For LHCP state, the current rotates in clockwise direction. Meanwhile, the vector current remains unchanged for the LP state.

### Design guideline

The design guidelines can be summarized as follows:

Step-1: Design the passive antenna with CP radiation

- Based on the method presented in^20^, design the radiating aperture consisting of four-square rings operating in the interested frequency band.
- Design a Y-shaped patch.
- Tuning the dimensions of the Y-shaped patch and the feeding position to achieve good performance in terms of reflection coefficient and AR.

Step-2: Design the reconfigurable feeding network

- Design a T-junction power divider working at the target frequency range.
- Inserting diodes and inductors into the divider.

Step-3: Design the polarization reconfigurable antenna

- Modify the Y-shaped patch in Step-1 with dual-feeding positions.
- Tuning the diode position to achieve the best impedance matching for the CP state.
- Tuning the dimensions of the excited patch and the feeding position to achieve good CP and LP operation.

## Measured results

**Fig. 10:**
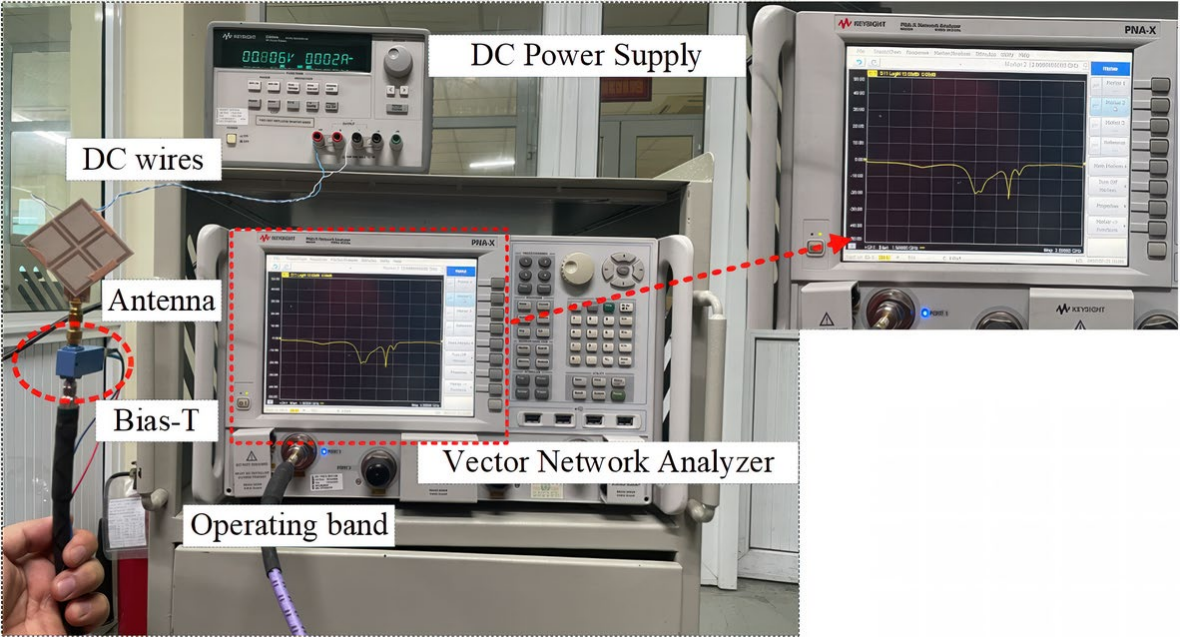
Photographs of the fabricated antennas.

To validate the practicality of the proposed concept, measurements are implemented on an fabricated prototype. Figure 10 displays photographs of the fabricated antenna along with the measurement setup incorporating a Bias-T. A Bias-T of type BT-28-400D is employed to link the SMA connector to the measurement cable, effectively preventing DC current from entering the VNA system^23^. The voltage is fine-tuned to ensure that the current through the diode remains approximately 30 mA. Overall, the measured data is sligltly different from the simulated data. As the proposed design has three different layers, the misalignment is unavoidable, and this results in the reason for this difference. Besides, an imperfection in measurement setup might be another reason.

**Fig. 11:**
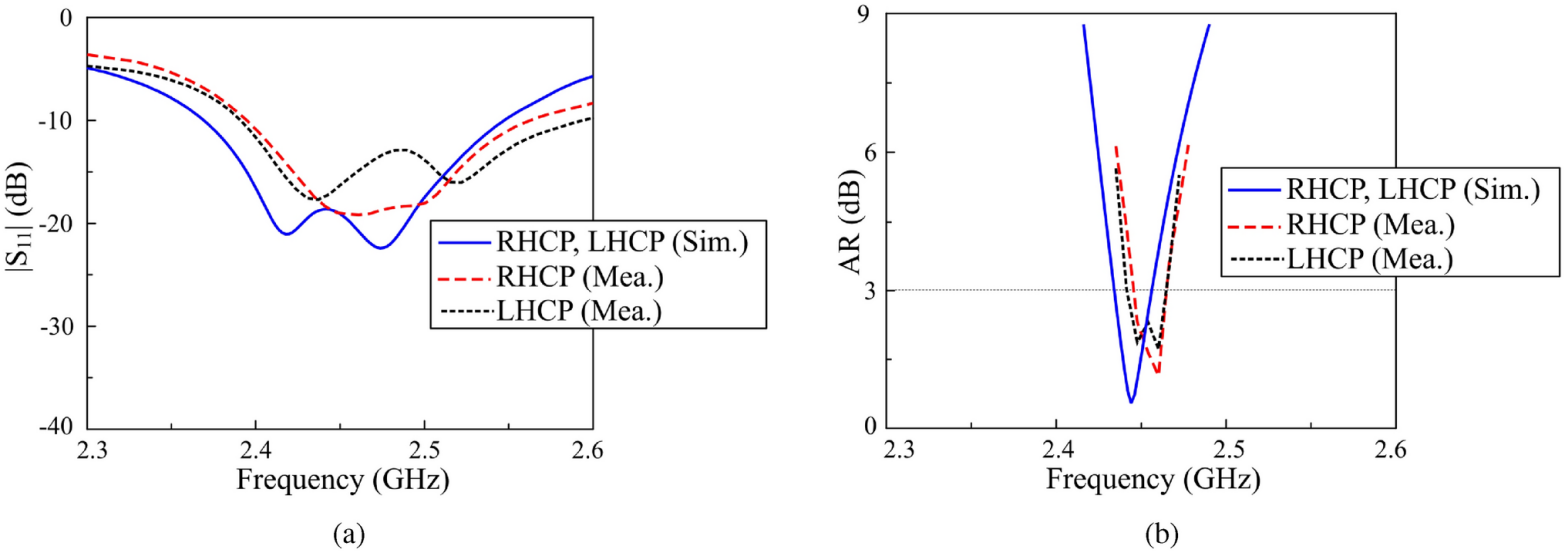
Simulated and measured results of (**a**) $|S_{11}|$ and (**b**) AR for the proposed antenna in dual-CP states.

**Fig. 12:**
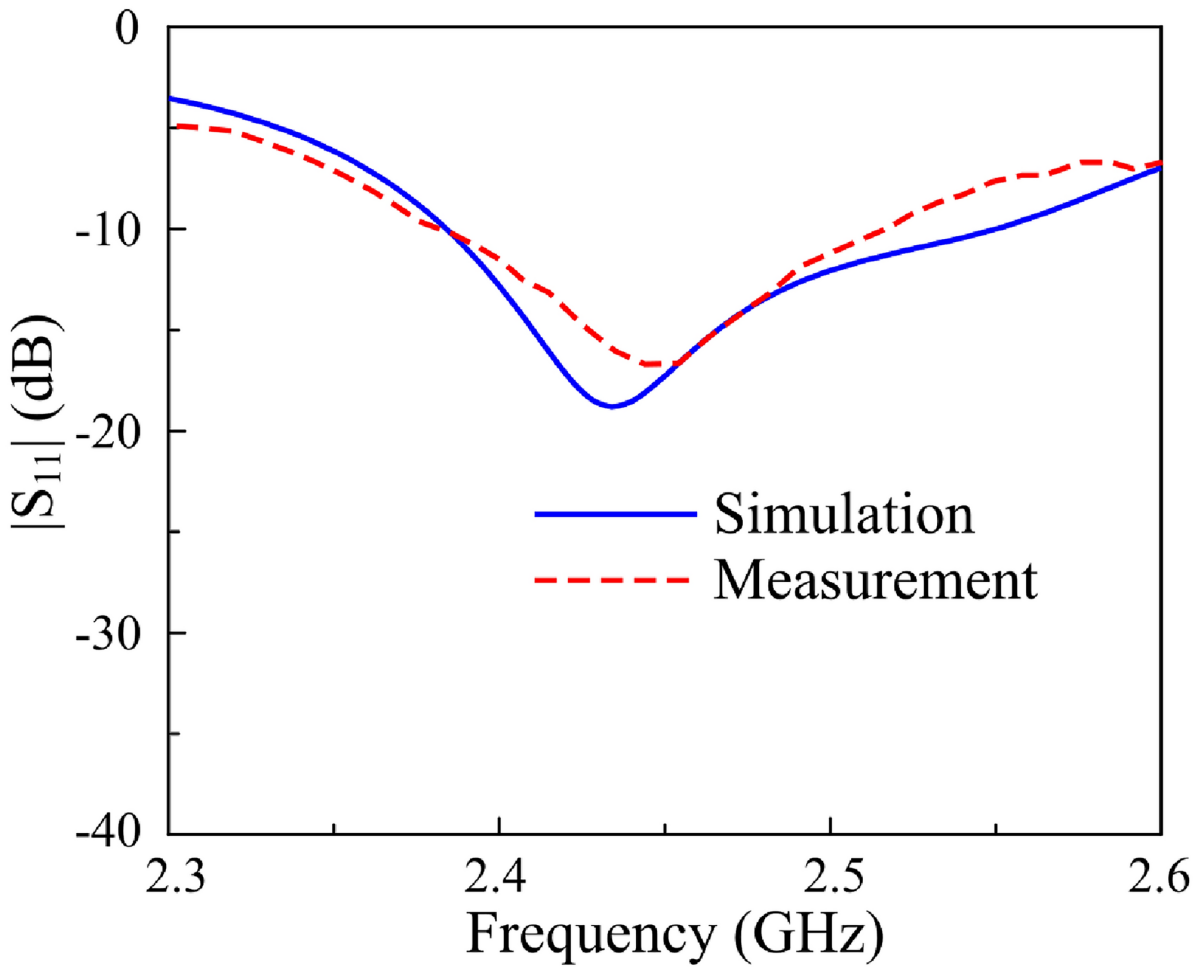
Simulated and measured $|S_{11}|$ for the proposed antenna in LP states.

**Fig. 13:**
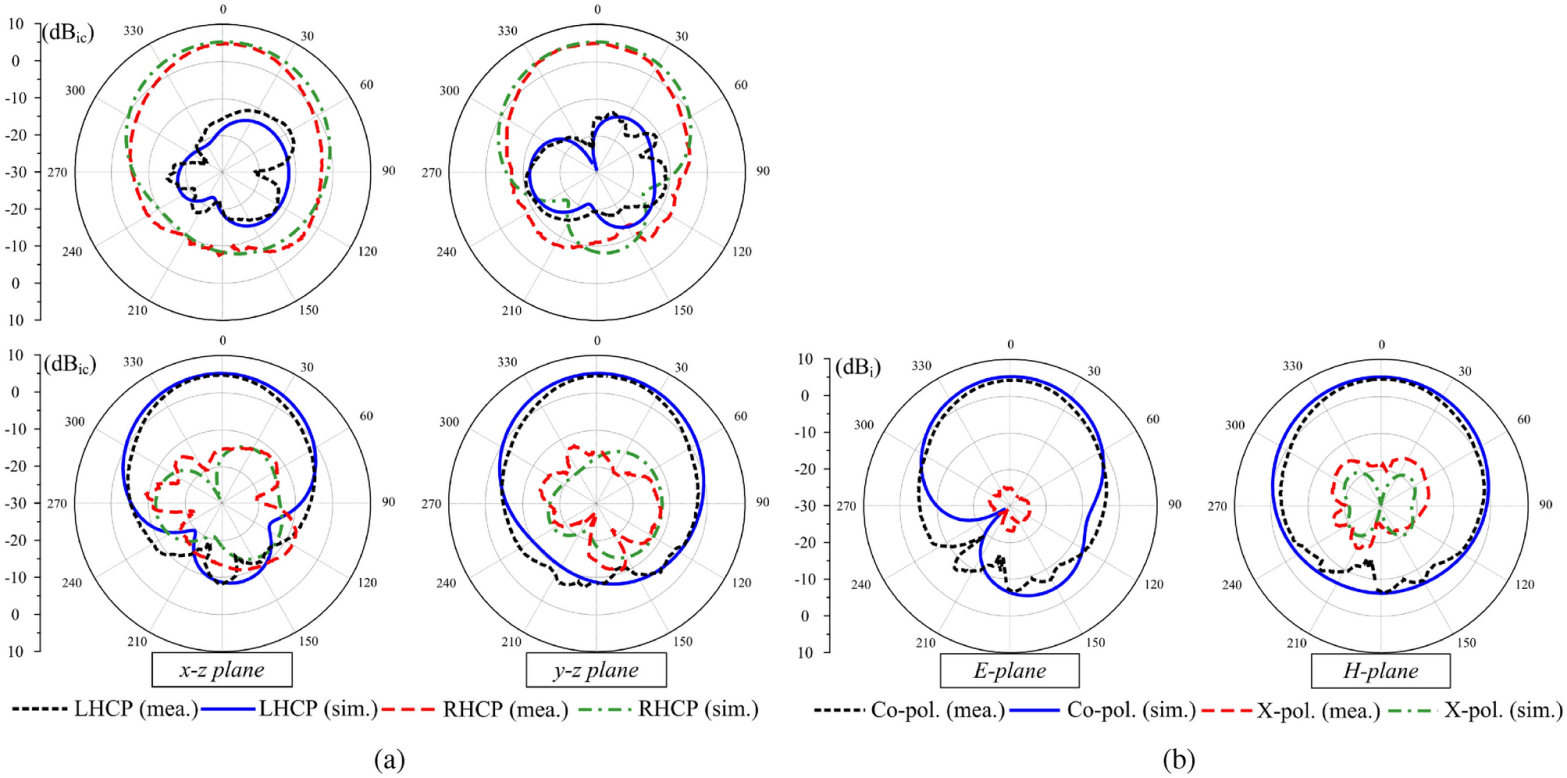
Simulated and measured radiation patterns at 2.45 GHz for (**a**) dual-CP and (**b**) LP states.

Figures 11 and 12 illustrate the simulated and measured $|S_{11}|$ and AR under different operating states. The measured overlapped bandwidth (BW) across all states ranges from 2.44 to 2.47 GHz. It is important to note that this bandwidth is defined such that the $|S_{11}|$ values remain below $-10$ dB for all states, while the AR values do not exceed 3 dB. The gain patterns in the two principal planes, namely *x*-*z* and *y*-*z*, are depicted in Fig. 13. The antenna exhibits a symmetric radiation pattern around the broadside direction, achieving a broadside gain of approximately 4.8 dBic. Additionally, the polarization isolation is around 18 dB, whereas the back radiation is reduced by 13 dB compared to the forward radiation.

## Performance comparison

Performance comparison among reconfigurable designs antennas is presented in Table 2. In this comparison, the complexity of the switching mechanism is evaluated based on the number of diodes utilized. It is evident that the proposed design benefits from a simplified switching mechanism, requiring the fewest diodes while still achieving three distinct polarization states. In terms of overall size, the proposed structure is the most compact. However, this comes at the cost of operating bandwidth (BW) and gain. Certain designs in^16–18^ achieve a wider operating bandwidth and higher gain due to the incorporation of high-profile structures and a greater number of radiating elements.

**Table 2:** Comparison among state-of-the-art polarization reconfigurable designs.

Ref	Overall size ($\lambda$)	No. of diodes	No. of states	Diode position	Bandwidth (%)	Peak gain (dBi)
^3^	$0.97 \times 0.97 \times 0.02$	2	2	Radiator	< 1.0	7.0
^4^	$0.57 \times 0.57 \times 0.02$	4	2	Radiator	3.6	2.9
^6^	$1.25 \times 1.25 \times 0.03$	4	2	Radiator	< 1.0	-
^7^	$0.58 \times 0.58 \times 0.02$	2	2	Radiator	2.0	3.2
^13^	$1.45 \times 1.45 \times 0.07$	2	3	Radiator	3.2	10.6
^14^	$1.23 \times 1.23 \times 0.03$	4	2	Radiator	1.7	6.2
^15^	$0.56 \times 0.56 \times 0.07$	10	2	Feeding network	6.4	7.2
^16^	$0.67 \times 0.67 \times 0.09$	8	2	Feeding network	20.8	6.9
^17^	$3.50 \times 1.33 \times 0.03$	3	3	Feeding network	1.8	12.2
^18^	$1.30 \times 1.30 \times 0.07$	4	3	Feeding network	17.8	9.4
Prop.	$0.36 \times 0.36 \times 0.03$	2	3	Feeding network	1.2	4.8

It is worth noting that the performance in terms of bandwidth and gain of the proposed antenna is still limited. Additionally, just one LP state is realized by the proposed design approach. The potential future research will focus on producing additional LP states and improving the operating bandwidth and gain.

## Conclusion

A polarization-reconfigurable antenna featuring a compact design and a straightforward switching mechanism has been introduced and analyzed in this paper. To minimize structural complexity, a reconfigurable feeding network with two PIN diodes and one T-divider has been employed. Additionally, the radiating aperture consists of four square-ring-shaped patches, which contribute to a reduced antenna size while maintaining favorable radiation characteristics. Measurements on the fabricated prototype validate that the proposed antenna effectively supports dual-CP and single-LP operations within the frequency range of 2.44 to 2.47 GHz, achieving a maximum gain of approximately 4.8 dBi.

## Data Availability

Data is provided within the manuscript.
